# Diagnosis and Methods of Repair for a Uteroperitoneal Fistula (UPF) Formed After Gynecological Surgeries

**DOI:** 10.7759/cureus.51064

**Published:** 2023-12-25

**Authors:** Nagisa Wada, Masato Tamate, Motoki Matsuura, Tsuyoshi Saito

**Affiliations:** 1 Obstetrics and Gynecology, Sapporo Medical University, Sapporo, JPN

**Keywords:** gynecological complication, hysteroscopy, fistula, infertility, laparoscopy

## Abstract

A uteroperitoneal fistula (UPF) is a rare disorder that can lead to infertility and has never been reported. UPFs can cause infertility and perinatal complications. A 34-year-old woman (gravida 0) with a history of three gynecological surgeries using a uterine manipulator was diagnosed with a UPF using hysteroscopy and hysterosalpingography. She underwent laparoscopic uterine repair as an infertility treatment. The uterine perforation may have been caused by uterine manipulator insertion or suture failure in the myometrium during her previous laparoscopic myomectomy and cystectomy procedures. The UPF disappeared after the current surgical treatment.

The complications of UPFs include infection, infertility, ectopic pregnancy, and uterine rupture. We expected that the presence of a fistula would increase the risk of impaired fertilization, implantation failure, and ectopic pregnancy. This case report contributes valuable insights into the diagnosis of UPFs and their laparoscopic repair.

## Introduction

A uteroperitoneal fistula (UPF) is an unreported clinical entity. Uterine surgeries may cause UPFs and we need to discuss asymptomatic UPFs. The patient detailed herein underwent three gynecological surgeries using a uterine manipulator, and the postoperative course was uneventful. However, several years after the surgeries, a UPF was identified during hysterosalpingography for infertility screening.

Uterocutaneous fistulas (UCFs) have been reported in the past [1]; however, the present case has never been reported and its etiology and optimal treatment remain unclear. The UPF may be a complication of uterine perforation during uterine surgeries. Uterine perforation is often detected intraoperatively and closed via repair. In rare cases, a perforation that does not heal may develop into a fistula. Pathophysiology of a UPF appears to be like a cesarean scar defect (CSD) [2] in which they have a notch in the uterine endometrium. A uterine notch may also reduce fertility. Theoretically, the accumulation of blood, mucus, and fluid in the notch and uterus may impair the penetration of sperm cells and the implantation of embryos [3]. Therefore, we considered it a possible cause of secondary infertility. We believe that the repair surgery performed in this asymptomatic UPF case can be applied in cases of uterine perforation and fistulas that occur in various uterine surgeries.

## Case presentation

The patient was 34 years old; she had had one pregnancy and zero births. She has been married for eight years and has been trying to conceive for seven years. She has a treatment history of endometriosis and uterine leiomyoma but no endocrine abnormalities.

**Figure 1:**
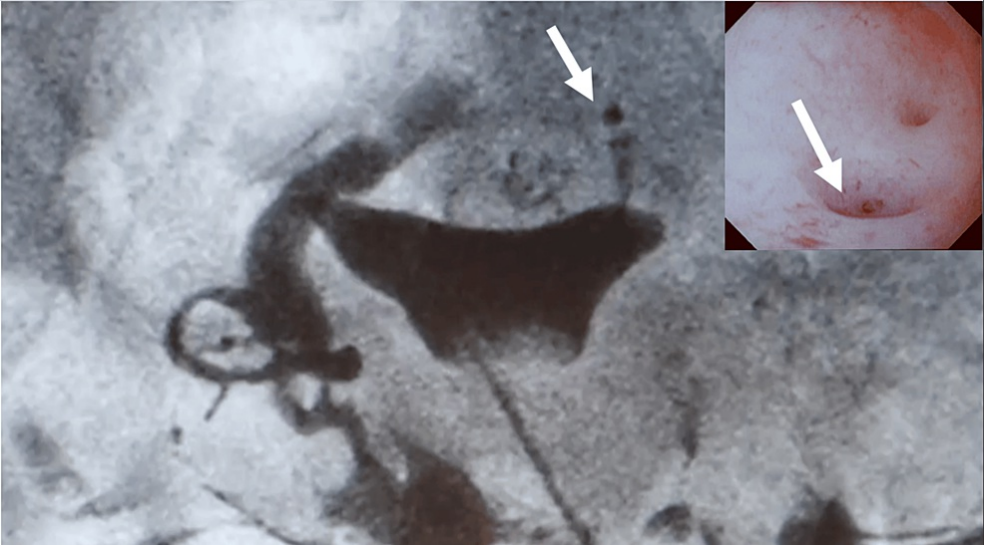
Preoperative hysteroscope and hysterosalpingography Arrows indicate niches in the uterine endometrium and leakage of contrast medium from the uterus into the abdominal cavity.

The patient had visited a nearby obstetrics and gynecology clinic for infertility treatment. She had no symptoms other than dysmenorrhea and her menstrual cycle was normal. Hysterosalpingography was performed for infertility screening, and a uterine fistula was suspected because of leakage of the contrast medium from the uterus (four months prior to presentation). Previously, she underwent laparoscopic-assisted ovarian cystectomy for ovarian endometriosis 11 years prior to presentation and laparoscopic-assisted ovarian cyst resection for the recurrence of ovarian endometriosis seven years prior to presentation. In addition, she had undergone laparoscopic uterine myomectomy and endometriosis ablation one year prior to presentation at the same hospital. Uterine manipulation devices were used in all the surgeries. The three myomas that were enucleated were separated from the endometrium and appeared to be intramural. The locations of these myomas corresponded to Federation Internationale de Gynecologie et d'Obstetrique (FIGO) classification O-4. They appeared to have been continuously sutured into the two layers of the myometrium. No damage to the endometrium was reported at the time of surgery. Hysteroscopy performed at our hospital revealed a notch/depression near the left oviductal opening, with saline outflow directed to the same site. Hysterosalpingography revealed leakage of the contrast medium from the posterior wall of the uterine fundus (Figure 1). Then, the patient was referred to our hospital for uterine repair.

**Figure 2:**
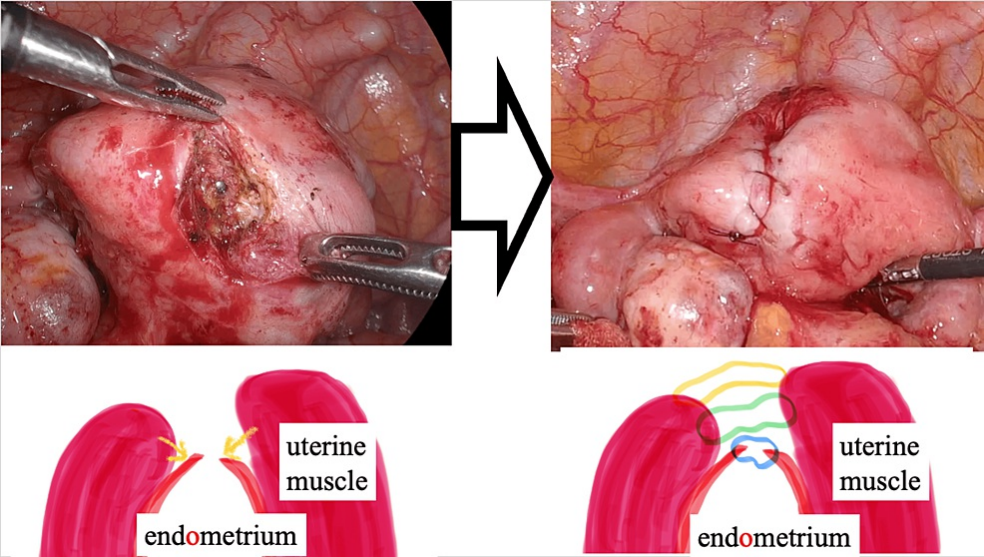
Procedure for laparoscopic uterine repair A sonde was inserted into the area where the dye fluid had leaked out to check the fistula area. The endometrium, myometrium, and serosa were each sutured.

Laparoscopic uterine repair was performed on the fistula on the left side of the uterine fundus to facilitate future fertility treatment, reduce the risk of complications during pregnancy and childbirth, and prevent intra-abdominal infections. We visualized the uterine fistula and thinned areas during laparoscopy using a hysteroscope and a light source, with reference to the rendezvous technique [4]. After identifying the thinning and extent of the fistula, we briefly clamped the fallopian tubes using forceps with a weak grasp and injected indigo carmine into the uterine lumen to enhance the visibility of the fistula. The left side of the uterine fundus was stained blue, suggesting thinning of the myometrium and the formation of a fine fistula. The fistula was approximately 2 mm in size, similar to the fallopian tube opening. A drop of water escaped, indicating a leak. An incision of approximately 1 cm was made vertically from the uterine serosa to the myometrium, and a margin was used to suture the endometrium and myometrium using 3-0 to 2-0 monofilament thread to cover the incisions. Complete excision of the tract, removal of the fistula tract, refreshment of the edges, and warm-saline wound irrigation were performed. The lack of saline outflow and the disappearance of the fistula were confirmed using a hysteroscope (Figure 2).

**Figure 3:**
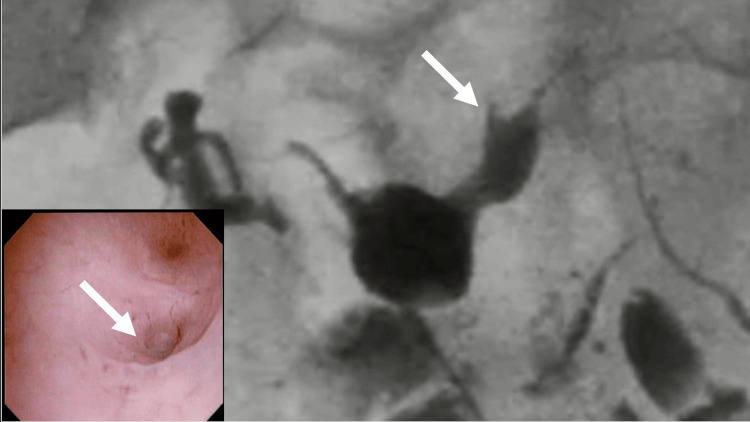
Postoperative hysteroscope and hysterosalpingography The arrows indicate that the contrast agent has disappeared from the uterus into the abdominal cavity.

No complications occurred during the postoperative period. In addition, hysterosalpingography conducted three months postoperatively showed no leakage of the contrast medium, confirming that the uterine fistula was repaired. Additionally, preoperative hysterosalpingography revealed no communication with the left fallopian tube; however, postoperative communication was confirmed (Figure 3). Pregnancy was permitted four months postoperatively, and infertility treatment was initiated and is currently in progress.

## Discussion

The UPF, a pathological communication between the uterus and intraabdominal peritoneum, is a rarely reported condition. We discuss the causes, diagnosis, and repair of an asymptomatic UPF. This case involved a UPF that developed after several surgeries and, therefore, may have been iatrogenic. Owing to the small size of the fistula, it is probable that the patient experienced no symptoms, such as abdominal pain. This was a rare case of a UPF diagnosed by hysterosalpingography. We believed that repairing the fistula would result in the following, even if the UPF is asymptomatic: (1) improving the reflux of menstrual blood; (2) reducing the risk regarding pregnancy in the form of causing the hole to widen owing to the loss of serosal membranes; and (3) reducing the risk of ectopic pregnancy in the fistula. Even if the fistula is small and all the layers from the myometrium to the serosa are missing, the semen could escape the fallopian tubes. In addition, the risk of holes spreading during pregnancy increases; therefore, closure is necessary.

Regarding the causes of UPFs, several reports have described the incidence of fistulae during uterine surgeries. However, these include UCFs and acquired vesicovaginal fistulae after cesarean section, myomectomy, or radiation therapy. Cases of intra-abdominal and uterine connection, as in the present case, are rare. In the present case, the laparoscopy-assisted ovarian cyst resection and laparoscopic myomectomy were performed by the same physician. Possible causes include uterine perforation during sonde and manipulator insertion as well as suture failure in the myometrium and serosa suture after uterine myomectomy. We speculated that the fistula was caused by retrograde menstruation after the uterine perforations [5]. Intra-abdominal infection, uterine bleeding, and uterine rupture during pregnancy are the expected complications of uterine perforation and uterine fistulas. In this case, future pregnancy was desired, and the increased risk of uterine rupture posed problems for future pregnancy and childbirth [6-8]. Without timely discovery and optimal repair of the uterine injury, all the aforementioned procedures can serve as triggers, leading to an abnormal opening from the uterine cavity, whereby abnormal excretion of menstrual blood and secretions to adjacent pelvic organs induce bridge adhesions, which provide physical support for fistula formation [1]. 

The hospital that performed the surgery informed us that the manipulator had penetrated close to the serosa. A history of myomectomies and severe damage to the endometrium caused by myoma excision and manipulator insertion appeared to be the major contributing factors to fistula formation in the current case. Additionally, uterine rupture may occur following intraoperative suture repair, wherein the uterus is perforated during the insertion of a laparoscopic manipulator [9]. This suggests that the perforated area was not closed, which increases the risk of rupture during pregnancy.

Regarding the diagnosis of UPFs, fistulograms, hysterosalpingography, and methylene blue tests can facilitate the diagnosis of UCFs. Magnetic resonance imaging and hysteroscopy are alternative methods for a definitive diagnosis in cases with a high suspicion of UCFs [10]. Assuming that the pathophysiology of UPFs is similar to that of UCFs and vesicouterine fistulas (VUFs), gonadotrophin-releasing agonists may serve as conservative therapy for UCFs; however, spontaneous healing is observed in only 50% (2/4) of the cases, and surgery is ultimately needed in the remainder, especially in cases where infertility treatment is desired [1]. 

In the current case, the endometrium was sutured using a single ligature suture, and the muscular layer and serosa were sutured; the key was to identify the layers. We believe that even if a fistula is repaired, there is still a risk of rupture after myomectomy; however, repair of the fistula can prevent the loss of all layers.

## Conclusions

Herein, we report a case of an asymptomatic UPF that developed after three surgeries and was revealed during hysterosalpingography for infertility screening. Uterine perforation is often reported as a complication of uterine surgery and intrauterine manipulation; however, clinicians need to be sufficiently aware of such complications and the management thereof. Regarding prevention, caution should be exercised when routinely inserting uterine manipulators. Although the UPF is asymptomatic, it should be diagnosed with contrast studies for patients who wish to have a pregnancy. If the patient is refractory to conservative treatment, we need to perform surgical treatment.

## References

[1] Han C, Zhang W, Li X, Sun B, Cheng L (2022). Postmyomectomy uterocutaneous fistula: a case report and literature review. Arch Gynecol Obstet.

[2] Riemma G, De Franciscis P, Torella M (2021). Reproductive and pregnancy outcomes following embryo transfer in women with previous cesarean section: a systematic review and meta-analysis. Acta Obstet Gynecol Scand.

[3] Vissers J, Klein Meuleman SJ, de Leeuw RA (2023). Effectiveness of laparoscopic niche resection versus expectant management in patients with unexplained infertility and a large uterine caesarean scar defect (uterine niche): protocol for a randomised controlled trial (the LAPRES study). BMJ Open.

[4] Nirgianakis K, Oehler R, Mueller M (2016). The Rendez-vous technique for treatment of caesarean scar defects: a novel combined endoscopic approach. Surg Endosc.

[5] Ibinaiye PO, Onwuhafua P, Usman B (2013). Utero-peritoneal fistula, a rare complication of laparoscopic myomectomy scar dehiscence: a case report. Niger Postgrad Med J.

[6] Koyama S, Kobayashi M, Tanaka Y, Isobe M, Miwa H, Shiki Y (2013). Laparoscopic repair of a post-myomectomy spontaneous uterine perforation accompanied by a bizarre tumor resembling polypoid endometriosis. J Minim Invasive Gynecol.

[7] Al-Zirqi I, Daltveit AK, Forsén L, Stray-Pedersen B, Vangen S (2017). Risk factors for complete uterine rupture. Am J Obstet Gynecol.

[8] Lee SJ, Ko HS, Na S (2020). Nationwide population-based cohort study of adverse obstetric outcomes in pregnancies with myoma or following myomectomy: retrospective cohort study. BMC Pregnancy Childbirth.

[9] Uccella S, Cromi A, Bogani G, Zaffaroni E, Ghezzi F (2011). Spontaneous prelabor uterine rupture in a primigravida: a case report and review of the literature. Am J Obstet Gynecol.

[10] Ilyas M, Khan I, Gojwari T, Dar MA, Shafi F, Shah OA (2019). Post-LSCS uterocutaneous fistula-utility of magnetic resonance imaging in its diagnosis. Turk J Obstet Gynecol.

